# Risk of Burnout in Danish GPs and Exploration of Factors Associated with Development of Burnout: A Two-Wave Panel Study

**DOI:** 10.1155/2013/603713

**Published:** 2013-12-07

**Authors:** Anette Fischer Pedersen, Christina Maar Andersen, Frede Olesen, Peter Vedsted

**Affiliations:** Research Unit for General Practice and Research Centre for Cancer Diagnosis in Primary Care (CaP), Health, Aarhus University, Bartholins Allé 2, 8000 Aarhus C, Denmark

## Abstract

*Background*. We assessed risk of burnout in GPs during a 7-year followup and examined whether (1) thoughts about changing medical specialty increased the risk of burnout and (2) burned out GPs had higher job turnover rates than burnout-free GPs. *Methods*. In 2004 and 2012, all GPs in the county of Aarhus, Denmark, were invited to participate in a survey. Retirement status of physicians who participated in 2004 was obtained through the Registry of Health Providers in 2012. *Results*. 216 GPs completed both surveys. The risk of developing burnout during the 7-year followup was 13.2% (8.2–19.6%). GPs who in 2004 were burnout-free and reported that they would not select general practice as medical specialty again had a statistically significant increased risk of burnout in 2012 (OR = 4.5; 95% CI = 1.2–16.5; *P* = 0.023). Among GPs with burnout in 2004, 25.0% had withdrawn from general practice during followup compared to 28.8% of burnout-free GPs in 2004 (adj. OR = 0.99; 95% CI = 0.48–2.02; *P* = 0.975). *Conclusion*. The 7-year incidence of burnout was 13%. Thoughts about changing medical specialty were an important predictor of burnout. Burned out GPs had not higher job turnover rates than burnout-free GPs.

## 1. Introduction

Burnout among general practitioners (GPs) appears to be common, with reported prevalence proportions varying from approximately 12% [1] to 42% [2]. The span in prevalence proportions may reflect actual differences in examined populations stemming from variations in demographic characteristics and in organization of general practice. Methodological issues such as differences in applied cutoffs for classification of burnout cases and cultural variations concerning the attitude to disclose poor well being may of course also play a role.

Burnout is a psychological construct defined as a prolonged response to chronic emotional and interpersonal stressors on the job and is characterized by emotional exhaustion, depersonalization, and a subjective experience of decreased personal accomplishment [3]. The negative effects of burnout on GPs' job performance are controversial. On the one hand, burnout has been associated with decreased empathy in medical students [4] and with an increased number of self-reported medical errors in surgeons [5]. On the other hand, two studies did not reveal significant associations between depersonalisation and patient-rated interpersonal skills [6] and between burnout symptoms and the GPs' awareness of patients' psychosocial problems [7].

Studies of burnout have often used a cross-sectional design and there is no good knowledge of the incidence and the risk of burnout. Taken the supposedly adverse effects of burnout into consideration, it becomes highly important to establish knowledge about the risk of burnout and gain insight into possible preventive initiatives. Various personal and occupational factors have been associated with burnout such as working hours, list size, and number of practice partners [8]. However, the causal interpretation is hampered due to cross-sectional study designs. Being married and having children have been shown to be associated with less burnout in GPs [9], but other studies have not supported such associations [2, 10, 11]. Likewise, results concerning the role of age and seniority are inconsistent [6, 10]. Women have fairly consistently been shown to report less depersonalization than men [6, 9, 12] but have also been shown to report higher levels of emotional exhaustion than their male colleagues [9].

The results of one cross-sectional study have revealed an association between the intention to change medical specialty and low job satisfaction [13]. While it seems likely that low job satisfaction can boost intentions to change specialty, the reverse causality would imply important perspectives. If wishing to change specialty is a precursor of burnout, this knowledge would be very important to, for example, colleagues and supervisors who guide GPs [14]. Furthermore, a few studies have shown that lower job satisfaction is linked to intentions to withdraw from general practice [13, 15, 16], but whether job turnover rates are actually higher in GPs with burnout compared to GPs without burnout is unknown.

On this background, the purpose of this study was to analyse (1) the risk of burnout among a sample of Danish GPs during a 7-year follow-up period, (2) whether thoughts about changing medical specialty increase the risk of burnout, and (3) whether the job turnover rates are higher for GPs with burnout than for GPs without burnout.

## 2. Methods

### 2.1. Setting

All GPs in Denmark are independent contractors with the regional health authorities, and they are fully responsible for the organization of the work in their practice. This also includes premises and staff. According to the national contract, the practice has to be open from 8 a.m. until 4 p.m., from Monday to Friday. Acute patients should be seen the same day and nonacute patients within five weekdays. Virtually all GPs make use of an appointment scheme with consultations of 10–15 minutes. Approximately 40% of GPs are solo GPs. The patient list size is on average 1550 patients per GP (including children) and 99% of citizens are registered with a particular general practice, which they have to consult. GPs act as gatekeepers to the rest of the health care system except for emergencies. GPs are remunerated on a mix of capitation and fee-for-service (25/75%).

### 2.2. Study Population and Surveys

In May 2004, all 458 active GPs in the county of Aarhus, Denmark, were invited to participate in a survey on job satisfaction, burnout, and working conditions (“the GP profile 2004”). The active GPs were identified by the Registry of Health Providers, which is managed by the National Board of Health. Nonrespondents were sent a reminder with a new questionnaire after four weeks, and GPs were remunerated with 16€ for responding. In January 2012, the survey on job satisfaction, burnout, and working conditions was repeated (“the GP Profile 2012”) and all active GPs in the Central Denmark Region (which since 2007 has included the former county of Aarhus) were invited to participate. Non-respondents were sent a reminder after four and thirteen weeks and GPs were remunerated in the amount of 50€ for responding.

According to Danish law, the study was not submitted to an ethical committee since questionnaire surveys do not require an ethical approval.

### 2.3. Questionnaire and Data

The questionnaire included a burnout scale and items about practice organisation (solo or group practice), provision of walk-in open access, weekly working hours in practice, monthly working hours in out-of-hour primary care, time used per consultation, and marital status for the GP. We used the Maslach Burnout Inventory Human-Services-Survey (MBI-HSS), which is considered to be a valid instrument for assessment of burnout symptoms [17]. The scale has been translated into Danish following standardised procedures. The MBI-HSS consists of 22 items and each item is scored on a 7-point Likert scale. The 22 items are divided into three subscales: (1) emotional exhaustion (9 items), (2) depersonalization (5 items), and (3) personal accomplishment (8 items). Each subscale receives a score which is categorised as low or high based on normative population score [17]. A high level of emotional exhaustion is defined as a score >26, and a high level of depersonalization is defined as a score >9. Low personal accomplishment is defined as a score <34 (reverse score). Although it has been argued that the MBI subscale scores should be treated as continuous data [17], a categorical approach has often been applied [1, 2, 9, 11, 12]. In this study, a moderate degree of burnout was defined as a high score on the emotional exhaustion subscale and/or a high score on the depersonalization subscale. A high score on the emotional exhaustion and depersonalization subscales and a low score on the personal accomplishment subscale are defined as a high degree of burnout [17].

Thoughts about leaving practice were assessed with one question: *would family medicine still be your choice if you were to select your medical specialty today?* The answers were “yes, I definitely would,” “yes, I probably would,” “neutral,” “no, I would probably not,” or “no, I would definitely not.” The answers “yes, I definitely would” and “yes, I probably would” were categorized as “yes” and the answers “no, I would probably not” or “no, I would definitely not” were categorized as “no.”

From the Registry of Health Providers, we received data on all the individual GPs in relation to date when starting in a specific practice, changing to another practice or stopping as GP. Data on age and gender was also included. From the National Health Insurance Registry, we got data on the number of listed patients per GP at May 1, 2004, and at February 1, 2012, and number of consultations provided per GP in 2003 and in 2011.

### 2.4. Analyses

GPs with a moderate and a high degree of burnout were collapsed. The incidence of burnout was estimated as the proportion of new cases of burnout in the 2012 survey to number of GPs who were burnout-free in 2004. We calculated the association between new cases of burnout and possible risk factors assessed in 2004, including sex, age, marital status, practice organisation, open access, weekly working hours in practice, monthly working hours in out-of-hour primary care, estimated time uses per consultation, number of enrolled patients, number of provided consultations, and the GPs' answer to the hypothetical question whether general practice would still be their choice if they were to select medical specialty today. The association was calculated as odds ratio (OR) in a logistic regression model. We calculated the crude OR and an adjusted OR controlling for the influence of sex and age. As some of the GPs were working in the same practice, analyses were corrected for clusters of GPs within the same practice using robust variance estimates. *P* values of 5% or less were considered statistically significant and data was analyzed using STATA 11.

## 3. Results

**Figure 1:**
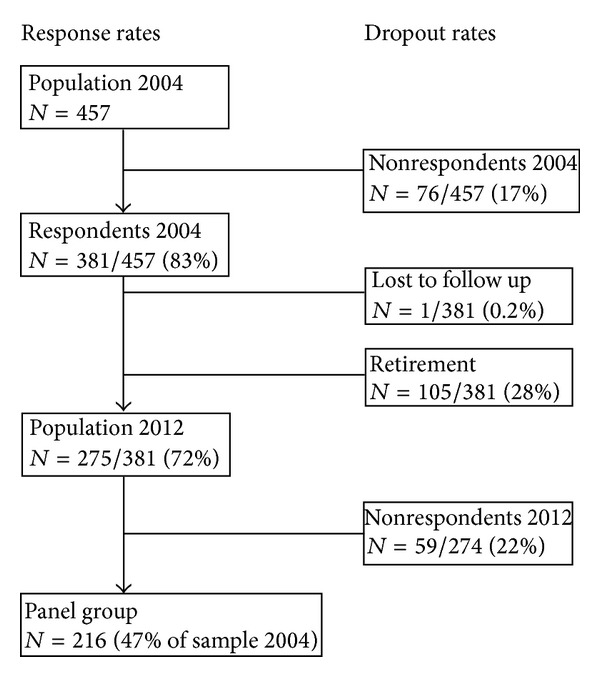
Response rates and dropout rates in the 2004 and 2012 surveys.

Figure 1 provides an overview of the response and dropout at the two measurement points. In this study, 105 (28%) were lost due to attrition in the period from May 2004 to January 2012. The panel group, that is, GPs who had completed both surveys, consisted of 216 GPs. The 105 retired GPs were older than the 216 GPs in the panel group as well as the 59 nonrespondents who did not complete the 2012 survey but were still active (*P* < 0.001). Characteristics of the 381 GPs who participated in the 2004 survey and the 216 GPs who participated in both surveys are shown in Table 1 stratified for gender.

The proportion of GPs indicating high levels of emotional exhaustion, depersonalization, and low levels of personal accomplishment is shown in Table 1. The proportion of female GPs reporting high emotional exhaustion had increased from 8.7% in 2004 to 18.6% in 2012. Among men the proportion had increased from 9.9% to 11.8%. The proportions of male and female GPs reporting high levels of depersonalization and low levels of personal accomplishment had decreased from 2004 to 2012 as the proportion of male GPs had reached the criteria for moderate burnout (from 25.0% in 2004 to 18.5% in 2012). Among females, the proportion of GPs with moderate burnout had increased from 20.8% to 23.7%. With respect to a severe degree of burnout, the proportion had increased slightly (2.6% in 2004 to 3.7% in 2012).

A total of 152 GPs were burnout-free in 2004 and in 2012; 20 new cases of moderate or severe degree of burnout were revealed, resulting in a risk of developing a moderate or high degree of burnout during the 7-year followup on 13.2% (95% CI: 8.2–19.6%). For male GPs, the risk was 11.9% (95% CI: 5.9–20.8%) and for women was 14.7% (95% CI: 7.3–25.4%; *P* = 0.637). Of those with burnout in 2004, 26 (40.6%) were without burnout in 2012.

Associations between new cases of burnout and possible risk factors assessed in 2004 are depicted in Table 2. GPs who were inconclusive or reported “no” to the question about choosing family practice as medical specialty again had a 4.5 (95% CI: 1.2–16.5, *P* = 0.023) risk of being a new case of burnout in the 2012 survey compared to GPs who answered that they would choose family practice as specialty again. None of the other included factors were significant predictors of burnout in 2012.

*Job Turnover Rates*. Twenty-two (25.0%) of the 88 GPs with burnout in 2004 had withdrawn from general practice during followup. In comparison, 78 (28.8%) of the 271 GPs who were burnout-free in 2004 had left general practice during followup. Adjusting for age and sex, there was no association between burnout status in 2004 and retirement rates during followup (OR = 0.99; 95% CI = 0.48–2.03; *P* = 0.975; data not shown).

## 4. Discussion

### 4.1. Key Findings

The risk of developing burnout in this sample of Danish GPs during the 7-year followup was 13.2%. A greater proportion of GPs who had developed burnout during the followup reported in 2004 that they were inconclusive or would not choose family medicine if they were to select medical specialty again compared to the proportion of GPs who were burnout-free in both surveys. Age and sex were not associated with the risk of developing burnout as were neither of the other risk factors examined. There was not a higher job turnover rates during followup in GPs who were burned out in 2004 than in GPs who were burnout-free in 2004. This indicates that GPs are able to or must stay in their practices until it is possible to retire.

### 4.2. Strengths and Limitations

Among the strengths of this study are the prospective design, use of an international validated scale for measuring burnout, and its high response rate. Although the study enjoyed a high response rate, a small group of GPs did not respond, which may have caused selection bias. It may be assumed that GPs experiencing burnout would have less energy and enthusiasm to complete the questionnaire. Insofar nonrespondents had a higher prevalence of burnout than respondents; the risk of burnout would have been underestimated. However, the opposite scenario could perhaps have been the case as some GPs suffering from high levels of burnout symptoms may have felt more inclined to participate in the study. One strength was the use of register based data which made it possible to make a nearly complete followup and to include data on the activity. The long followup strengthens this study since burnout is assumed to be a condition which takes time to develop. However, as many of the included variables are alterable, more assessment points during followup would have benefitted the study. For instance, it cannot be excluded that we have missed some incident cases of burnout during followup if the afflicted GPs had recovered before the 2012 survey.

### 4.3. Comparison with Existing Literature

A study of first-year internal medicine residents from five different training programmes in US yielded a risk of burnout during a 1-year followup on 75% [11]. The much higher burnout risk obtained in this study compared to the risk obtained in our study occurred despite fairly consistent definitions of burnout. Thus, the difference could be an indication of the influence of experience and age, as the study populations consisted of trainees and specialist doctors, respectively.

To our knowledge, the observed strong association between being reserved about choosing family practice as medical specialty again and increased risk of burnout has not been reported before. Thoughts about switching specialty may indicate a poor match between the personality of the GP and the job demands. This falls in line with Maslach and Leiter's model proposing that the greater the perceived mismatch between the person and the job, the greater the likelihood of burnout [18]. The findings of this study suggest that the hypothetical question about whether one would select the same medical specialty again may be used as a screening tool to identify GPs in risk of developing burnout.

Even though low levels of job satisfaction and high levels of perceived job-related pressure have been associated with intentions to leave general practice [13, 15, 16], we did not find a higher job turnover in GPs with burnout compared to GPs who were burnout-free in 2004. Thus, when a burned out GP discloses an intention to leave general practice, it may reflect a state of despair, but in reality leaving practice or “giving it up” may act against the personality of the burned out GP often characterized by high levels of conscientiousness [19] and a performance-based self-esteem [20].

Congruent with previous findings [6, 21], we found nonsignificant tendencies that the prevalence of burnout was higher in younger GPs than in older GPs and lower in GPs in solo practices than in GPs in other types of practices.

It has been documented fairly consistently that female physicians report lower levels of depersonalization and higher levels of emotional exhaustion than male physicians [6, 9, 12], and our results supported this.

## 5. Conclusions

The risk of developing a moderate or high degree of burnout in this sample of Danish GPs during the 7-year followup was 13.2%. GPs who in 2004 reported that they would not choose family medicine if they were to select medical specialty again or were inconclusive about this issue more than four-doubled their risk of burnout in 2012 compared to GPs whose choice of medical specialty would still be family medicine. We suggest more research into whether this particular item can be used as a screening tool among GPs to identify GPs at risk for developing burnout whereby an adjuvant intervention would be possible. Burned out GPs appear not to have higher job turnover rates than burnout-free GPs.

## Figures and Tables

**Table 1:** Characteristics of the 381 GPs who participated in the 2004 survey and the 216 GPs who participated in both surveys (the panel group).

	2004 *N* (%)			2012 *N* (%)		
	All 381 (100)	Men 232 (60.9)	Women 149 (39.1)	All 216 (100)	Men 119 (55.1)	Women 97 (44.9)
Age						
<45	60 (15.8)	22 (9.5)	38 (25.5)	2 (0.9)	2 (1.7)	0 (0.0)
45–49	87 (22.8)	43 (18.5)	44 (29.5)	28 (13.0)	11 (9.2)	17 (17.5)
50–54	81 (21.3)	48 (20.7)	33 (22.2)	57 (26.4)	23 (19.3)	34 (35.1)
55–59	102 (26.8)	75 (32.3)	27 (18.1)	55 (25.5)	30 (25.2)	25 (25.8)
>59	46 (12.1)	40 (17.2)	6 (4.0)	67 (31.0)	49 (41.2)	18 (18.6)
Missing information	5 (1.3)	4 (1.7)	1 (0.7)	7 (3.2)	4 (3.4)	3 (3.1)
Marital status						
Married	347 (91.1)	217 (93.5)	130 (87.3)	193 (89.4)	109 (91.6)	84 (86.6)
Single	27 (7.1)	12 (5.2)	15 (10.1)	20 (9.3)	10 (8.4)	10 (10.3)
Missing information	7 (1.8)	3 (1.3)	4 (2.7)	3 (1.4)	0 (0.0)	3 (3.1)
Practice organisation						
Solo practice	92 (24.1)	76 (32.8)	16 (10.7)	33 (15.3)	26 (21.9)	7 (7.2)
Group practice	33 (8.7)	25 (10.8)	8 (5.4)	33 (15.3)	25 (21.0)	8 (8.3)
Shared practice	94 (24.7)	42 (18.1)	52 (34.9)	54 (25.0)	23 (19.3)	31 (31.7)
Partnership practice	147 (38.6)	81 (34.9)	66 (44.3)	93 (43.1)	44 (37.0)	49 (50.5)
Missing information	15 (3.9)	8 (3.5)	7 (4.7)	3 (1.4)	1 (0.8)	2 (2.1)
Open access						
Yes, every day	32 (8.4)	22 (9.5)	10 (6.7)	37 (17.1)	23 (19.3)	14 (14.4)
Yes, but less than every day	21 (5.5)	13 (5.6)	8 (5.4)	13 (6.0)	5 (4.2)	8 (8.3)
No	328 (86.1)	197 (84.9)	131 (87.9)	166 (76.9)	91 (76.5)	75 (77.3)
Weekly patient-related working hours in practice						
<37	119 (31.2)	60 (25.9)	59 (39.6)	96 (44.4)	49 (41.2)	47 (48.5)
37–41	126 (33.1)	81 (34.9)	45 (30.2)	59 (27.3)	32 (26.9)	27 (27.8)
42–46	80 (21.0)	51 (22.0)	29 (19.5)	29 (13.4)	19 (16.0)	10 (10.3)
47–51	40 (10.5)	26 (11.2)	14 (9.4)	16 (7.4)	10 (8.4)	6 (6.2)
>51	11 (2.9)	11 (4.7)	0 (0.0)	6 (2.8)	2 (1.7)	4 (4.1)
Missing information	5 (1.3)	3 (1.3)	2 (1.3)	10 (4.6)	7 (5.9)	3 (3.1)
Monthly working hours in out-of-hour primary care						
0	144 (37.8)	81 (34.9)	63 (42.3)	110 (50.9)	59 (49.6)	51 (52.6)
1–24	125 (32.8)	75 (32.3)	50 (33.6)	79 (36.6)	41 (34.5)	38 (39.2)
>24	89 (23.4)	66 (28.5)	23 (15.4)	21 (9.7)	17 (14.3)	4 (4.1)
Missing information	23 (6.0)	10 (4.3)	13 (8.7)	6 (2.8)	2 (1.7)	4 (4.1)
Estimated time used per consultation						
≥13 min	295 (77.4)	169 (72.8)	126 (84.6)	177 (81.9)	93 (78.2)	84 (86.6)
<13 min	81 (21.3)	60 (25.9)	21 (14.1)	38 (17.6)	25 (21.0)	13 (13.4)
Missing information	5 (1.3)	3 (1.3)	2 (1.3)	1 (0.5)	1 (0.8)	0 (0.0)
Number of patients per GP						
<1226	97 (25.5)	47 (20.3)	50 (33.6)	46 (21.3)	23 (19.3)	23 (23.7)
1226–1620	192 (50.4)	115 (49.6)	77 (51.7)	113 (52.3)	59 (49.6)	54 (55.7)
>1620	92 (24.2)	70 (30.2)	22 (14.8)	57 (26.4)	37 (31.1)	20 (20.6) )
Number of consultations per GP in 2003						
<3588	92 (24.2)	45 (19.4)	47 (31.5)	51 (23.6)	23 (19.3)	28 (28.9)
3588–4993	195 (51.2)	115 (49.6)	80 (53.7)	114 (52.8)	58 (48.7)	56 (57.7)
>4993	94 (24.7)	72 (31.0)	22 (14.8)	51 (23.6)	38 (31.9)	13 (13.4
General practice would still be your choice if you were to select medical specialty today						
Yes	323 (84.8)	188 (81.0)	135 (90.6)	183 (84.7)	103 (86.6)	80 (82.5)
Neutral	30 (7.9)	23 (9.9)	7 (4.7)	22 (10.2)	12 (10.1)	10 (10.3)
No	21 (5.5)	16 (6.9)	5 (3.4)	7 (3.2)	3 (2.5)	4 (4.1)
Missing information	7 (1.8)	5 (2.2)	2 (1.3)	4 (1.9)	1 (0.8)	3 (3.1)
Emotional exhaustion						
Low emotional exhaustion	339 (89.0)	205 (88.4)	134 (89.9)	181 (83.8)	104 (87.4)	77 (79.4)
High emotional exhaustion	36 (9.5)	23 (9.9)	13 (8.7)	32 (14.8)	14 (11.8)	18 (18.6)
Missing information	6 (1.6)	4 (1.7)	2 (1.3)	3 (1.4)	1 (0.8)	2 (2.1)
Depersonalisation						
Low depersonalisation	308 (80.8)	183 (78.9)	125 (83.9)	192 (88.9)	105 (88.2)	87 (89.7)
High depersonalisation	66 (17.3)	44 (19.0)	22 (14.8)	22 (10.2)	13 (10.9)	9 (9.3)
Missing information	7 (1.8)	5 (2.2)	2 (1.3)	2 (0.9)	1 (0.8)	1 (1.0)
Personal accomplishment						
High pers. accomplishment	226 (59.3)	130 (56.0)	96 (64.4)	157 (72.7)	85 (71.4)	72 (74.2)
Low pers. accomplishment	137 (36.0)	93 (40.1)	44 (29.5)	55 (25.5)	32 (26.9)	23 (23.7)
Missing information	18 (4.7)	9 (3.9)	9 (6.0)	4 (1.9)	2 (1.7)	2 (2.1)
Moderate degree of burnout	89 (23.4)	58 (25.0)	31 (20.8)	45 (20.8)	22 (18.5)	23 (23.7)
Severe degree of burnout	10 (2.6)	7 (3.0)	3 (2.0)	8 (3.7)	5 (4.2)	3 (3.1)

**Table 2:** Association between new cases of burnout in the 2012 survey and possible risk factors (*N* = 152).

Variables assessed in the 2004 survey	*N*	New cases of burnout (%)	Univariate			Multivariate^a^		
			OR	95% CI	*P* value	OR	95% CI	*P* value
Marital status								
Married	142	13.4	1	(Ref)		1	(Ref)	
Single	9	11.1	0.8	0.1–7.0	0.848	0.8	0.1–10.6	0.897
Practice org.								
Other than solo	114	13.2	1	(Ref)		1	(Ref)	
Solo	33	9.1	0.7	0.2–2.4	0.531	0.8	0.2–3.2	0.702
Open access								
Weekly or never	143	12.6	1	(Ref)		1	(Ref)	
Daily	9	22.2	2.0	0.4–9.3	0.385	2.3	0.5–10.9	0.311
Weekly patient-related working hours in practice								
<42	106	13.2	1	(Ref)		1	(Ref)	
≥42	46	13.0	1	0.4–2.6	0.977	1.1	0.4–2.9	0.815
Monthly working hours in out-of-hour primary care								
0	53	13.2	1	(Ref)		1	(Ref)	
1–24	49	12.2	0.9	0.3–2.7	0.877	0.6	0.2–2.0	0.392
>24	46	13.0	1.0	0.3–3.1	0.980	0.8	0.2–2.5	0.681
Minutes per cons.								
≥13 min	120	12.5	1	(Ref)		1	(Ref)	
<13 min	32	15.6	1.3	0.5–3.6	0.619	1.4	0.4–4.4	0.614
Number of patients per GP								
≥1226	118	11.9	1	(Ref)		1	(Ref)	
<1226	34	17.7	1.6	0.6–4.4	0.365	1.8	0.6–5.3	0.287
Number of consultations per GP in 2003								
≤4993	119	13.5	1	(Ref)		1	(Ref)	
>4993	33	12.1	0.9	0.3–2.8	0.841	0.9	0.2–3.4	0.892
General practice would still be your choice if you were to select your medical specialty today								
Yes	136	11.0	1	(Ref)		1	(Ref)	
No or neutral	16	31.3	3.7	1.1–12.1	0.032	4.5	1.2–16.5	0.023
Sex								
Male	84	11.9	1	(Ref)				
Female	68	14.7	1.3	0.5-3.3	0.616			
Age								
<55	120	15.0	1	(Ref)				
≥55	32	6.3	0.4	0.1–1.7	0.212			

^a^The multivariate analyses adjusted for sex, age, and baseline scores (2004) on emotional exhaustion, depersonalization, and personal accomplishment. Analyses are corrected for clusters of GPs within the same practice.
